# Avermectin induces the oxidative stress, genotoxicity, and immunological responses in the Chinese Mitten Crab, *Eriocheir sinensis*

**DOI:** 10.1371/journal.pone.0225171

**Published:** 2019-11-25

**Authors:** Yi Huang, Yuhang Hong, Zhiqiu Huang, Jilei Zhang, Qiang Huang

**Affiliations:** Key Laboratory of Animal Disease Detection and Prevention in Panxi District, Xichang University, Xichang, Sichuan Province, China; Zhejiang University College of Life Sciences, CHINA

## Abstract

Avermectin is commonly used in aquaculture systems for pest control in recent decades in China. However, no information is provided for the toxic effect to the important commercial species, Chinese mitten crab, *Eriocheir sinensis*. To investigate the aquatic toxicity of avermectin, an acute toxic test was performed in this study. The results showed that the 48 h- and 96 h- LC_50_ were 1.663 and 0.954 mg/L, respectively. For further research, crabs were exposed to sublethal concentrations of 0.03, 0.06, 0.12, 0.24 and 0.48 mg/L. Levels of antioxidants, including superoxide dismutase (SOD), catalase (CAT) and total antioxidant capacity (T-AOC) were significantly (P<0.05) decreased with dose- and time- dependent responses, meanwhile the oxidative products including malondialdehyde (MDA), hydrogen peroxide (H_2_O_2_) and protein carbonyl in serum increased significantly (P<0.05) at concentrations of 0.24 and 0.48 mg/L throughout the experiment. A significant (P<0.05) increase of intracellular ROS and decrease of phagocytic activity was observed in high concentration groups, with dose- and time- dependent manners during the exposure. In addition, serious genetic damage was detected, for the significant increase (P<0.05) of both comet ratio and %DNA in tail at each concentration, and micronucleus (MN) frequency at concentrations of 0.12, 0.24 and 0.48 mg/L at 96 h. These results indicated that sublethal concentration exposure of avermectin had a prominent toxic effect on *E*. *sinensis* based on the oxidative stress induced by generated ROS, immunological activity inhibition and genotoxicity.

## Introduction

Pesticide plays an important role in modern agriculture for its efficiency of production increase by pest control. However, a pesticide pollution in environment has became the major concern due to their extensive use in agriculture in recent decades. Avermectins (AVMs), a macrocyclic lactone compound, was first discovered in the extracts of the fungus *Streptomyces avermitilis* [1]. Avermectin B1, the main component of AVM, has been widely used as a pesticide to control agricultural pests or as anthelmintic against both internal and external parasites of livestock [2]. Avermectin family mainly act through the glutamate and γ-amino butyric acid (GABA)-gated chloride channel, which is located in brain cells and secured by the blood-brain barrier in mammals [3,4]. As consequence, avermectin is regard as relatively safe for human and other vertebrates, but it’s potential ecological risks for non-target species and food chain contamination need more attention. Avermectin is widely utilized in crop production including tree fruits, nuts, rice and cottons since it came into china in 1991. In the mean time, China has became the top producer of avermectin with an annual output of more than 3500 tons, with extensive use in both agricultural and non-agricultural area, such as aquatic ponds for pest control in fish breeding [5]. The ecological toxic effects of avermectin on aquatic organisms were often ignored by practitioners because it is not likely to be detected in high concentrations in water for the relatively short half-life [6]. However, numerous reports have shown that avermectin is highly toxic for some aquatic species, especially microcrustaceans and fish [7,8,2,9].

The Chinese mitten crab, *Eriocheir sinensis*, is one of the most important fresh water species widely bred in China with an annual output of more than 850,000 tons. Rice-crab co-culture is a high benefit eco-breeding pattern and has widespread developed in east China in recent decades [10]. As a bottom dweller, *E.sinensis* may be more vulnerable in avermectin exposure than fish due to the strong binding of the pesticide to soil and sediments [11]. From our observation in field, crabs climbed to the shore of the pond in a co-culture system after AVM spraying, which indicating the environmental stress response in animals. Therefore, we speculated that avermectin may be highly toxic to *E.sinensis*. However, study on the toxic effect of avermectin on this commercially important species is scarce at present. It is therefore urgent to investigate the toxic effect of avermectin at environmental related concentrations to *E*. *sinensis*.

Environmental stressors including pesticide can cause oxidative stress by inducing generation of reactive oxygen species (ROS). ROS may cause damage to DNA and biological macromolecules to induce cell injury [12]. Consequently, the antioxidant defences are potentially sensitive biomarkers to assess the pesticides exposure. In the present study, several commonly used parameters [13] including superoxide dismutase (SOD), catalase (CAT), total antioxidant capacity (T-AOC), malondialdehyde (MDA), hydrogen peroxide (H_2_O_2_) and protein carbonyl (PC) were measured, to evaluate the oxidative stress during sublethal exposure of avermectin. Moreover, ROS could alter protein structure or function and amino acid side-chains can be irreversibly modified into aldehyde or ketone groups (carbonylation) [14], hence, the level of protein carbonyl was also detected in this study to clarify the relevance between oxidative stress and tissue injury. In addition, previous studies demonstrated that avermectin could induce genotoxic damage by chromatin condensation and DNA fragmentation in both human and insect cells [15,16]. Therefore, the comet assay, which is usually performed to assess DNA damage from environmental pollution in aquatic animals, was conducted in the present study, combining with a micronucleus (MN) assay to investigate the genotoxicity of avermectin.

Overall, avermectin or its family members can induce oxidative and immunological damage as well as genotoxicity in mammals, birds and fish; however, studies of toxic effects of avermectin on freshwater crabs are rare. Besides, the main factors which induce oxidative stress in crab under the pesticides exposure are unknown. We hypothesis that avermectin exposure may induce ROS generation and oxidative stress, leading to immunological damage and genotoxicity in crabs. Our study could help in understanding the biological response of *E.sinensis* after avermectin exposure, and provide a reference for further research.

## Methods and materials

### Animals and chemicals

Adult crabs, *Eriocheir sinensis* (Crustacean: Decapoda: Grapsidae), with average weight of 92 ± 11 g were collected from a commercial farm in Shanghai, China. Animals were kept in a recirculation system with 12h: 12h photoperiod for at least 2 weeks for acclimatization. Animals were fed once a day at 7:00 p.m with commercial crab ration, and residuals and faeces were removed two hours after feeding. Crabs without any amputated limbs were chosen for the experiment. All experimental protocols were reviewed and approved by the Animal Bioethics Committee, Xichang University, China. Avermecin (AVM B1a, > 90% pure, CAS # 71751-41-2) used in this test was purchased from Sigma-Aldrich Chimicals (Germany) and acetone (analytical standard) was purchased from Sinochem Crop Care Co. LTD (Shanghai).

### Median lethal concentration (LC_50_) of avermectin on *E*. *sinensis*

A static acute toxicity test was conducted for the determination of LC_50_ of avermectin. Firstly, avermectin was dissolved in acetone at 10 mg/mL as a stock solution, and then diluted to concentrations of 0.50, 0.79, 1.26, 2.00, 3.16 and 5.00 mg/L in 75 cm × 45 cm × 60 cm glass aquarium with 50 L aerated tap water, and a control group without any avermectin was carried out in this experiment. Group of 5 mg/L received the highest concentration of acetone with 0.05%, which was below the no observed effect concentration (NOEC) of 0.1% reported by Mayer [17] and Johnson [18]. As a consequence, no solvent control was set up. There were four replicates for each treatment with 10 crabs and totally 240 crabs were used. During the experiment, water conditions were monitored daily and shown as follows: temperature 25 ± 1.0°C, pH 7.3 ± 0.6, dissolved oxygen 6.8 ± 0.4, conductivity 245 μS/cm, total hardness 202 mg/L as CaCO_3_, ammonia nitrogen<0.2 mg/L, and nitrite<0.005 mg/L. To avoid rapid photodegradation of avermectin, the illumination on the surface of aquarium was controlled to 300 lux. Animals were checked every 2 h and dead crabs were identified by no movement of appendages or eyestalks in response to a stick poke and were removed immediately. The 48 h- and 96 h- LC_50_ and 95% confidence limits were calculated by a regression probit analysis method in SPSS V21.0 software.

### Sublethal concentration exposure

According to the 48 h- and 96 h- LC_50_ values in the test above and the application dosages in a crop farm [9] and a rice field [19], five concentration groups (0.03, 0.06, 0.12, 0.24, and 0.48 mg/L) were set in the exposure experiment. They represent 1/32, 1/16, 1/8, 1/4 and 1/2 of the 96 h- LC_50_ values respectively. In addition, a group without any avermectin was treated as control. Each group was replicated three times with 10 crabs, and a total of 180 crabs were used. Water in the aquarium was aerated constantly and renewed half a day by adding fresh water containing the same concentration of avermectin. Water conditions were monitored and maintained the same as described above. Based on the research from Zhang et al that 87.2% of avermectin remained after 12 h exposure in a aquarium under the same environmental conditions (T = 25°C, pH 7.2–7.5, illumination 300 lux) [20], no measurement of the level of avermectin in water was carried out in the present study, and concentrations of avermectin in this test was represented as nominal concentrations.

### Sampling

Four individuals were randomly taken from each treatment at 0, 24, 48, 72 and 96 h following the exposure. Haemolymph was drawn with a sterile 1-mL syringe from the unsclerotised membrane of walking legs. Some haemolymph was diluted 1:1 with sterile anticoagulant (30 mM of trisodium citrate, 338 mM of NaCl, 115 mM of glucose, and 10 mM of EDTA) immediately for the MN assay, and some was centrifuged at 1200 r/min for 5 min to collect haemocytes, for the ROS detection, phagocytic activity assay and comet assay. The remaining haemolymph was used for subsequent assays of oxidative stress parameters including SOD, CAT, T-AOC, MDA, H_2_O_2_ and protein carbonyl.

### Antioxidant activities

Haemolymph was centrifuged at 11,000 r/min at 4°C for 30 min to collect supernatant as serum. SOD and CAT activities were determined by a spectrophotometric method at 550 nm and 405 nm respectively with the corresponding detection kits (Nanjing Jiancheng Bioengineering Institute, China) according to the manufacturer's protocols. One unit of SOD was defined as the enzyme activity that inhibited the photoreduction of NBT to blue formazan by 50% and was expressed as U/mL. One unit of CAT activity was defined as the amount of enzyme that catalysed the decomposition of 1 mmol of H_2_O_2_ per min and was expressed as U/mL. T-AOC was measured at 593 nm with the ferric reducing ability of plasma (FRAP) assay [21].

### Oxidative parameters

Oxidative damage was represented as lipid peroxidation (LPO) products which was indirect detection from thiobarbituric acid reactive substance (TBARS) (measured as MDA) and by reactive oxygen species (measured as H_2_O_2_). MDA and H_2_O_2_ in serum were detected spectrophotometrically at 532 and 405 nm with corresponding detection kits (Nanjing Jiancheng Bioengineering Institute, China) according to the manufacturer's protocols. The levels of MDA and H_2_O_2_ were quantified by the reaction with 2-thiobarbituric acid (TBA) and molybdic acid respectively and expressed as nmol/L. In addition, an ultraviolet spectrophotometric method was conducted at 370 nm with a detection kit (Nanjing Jiancheng Bioengineering Institute, China) to investigate the level of protein carbonyl in haemolymph. The protein carbonyl level was expressed as nmol carbonyl per mg of protein. The protein content in samples was determined spectrophotometrically according to the previous method [22] by using bovine serum albumin as a standard.

### Intracellular ROS detection

After the sampling of haemocytes, the production of ROS in cells was detected by using a ROS Assay Kit (Beyotime, China) with the DCFH-DA oxidation method [15]. Haemocytes were resuspended in L-15 medium (without serum) with 10 μM DCFH-DA and incubated at 28°C for 20 min to allow the fluorescent probe to diffuse into the cells. Then, cells were washed twice in PBS to remove extracellular DCFH-DA. The generation of ROS in cells was observed under an inverted fluorescence microscope (DM-i8, Leica, Germany) with wavelengths of excitation of 488 nm and emission of 525 nm. To evaluate the level of ROS, a semi-quantitative method was adopted by using Image J software (USA).

### Phagocytic activity

The phagocytic activity of haemocytes was detected by the method described in previous study [23]. Haemocytes were mixed with yellow-green fluorescent latex beads (particle size: 1 mm, density: 4.57 ×10^8^ per milliliter, Sigma), seeded into 24-well culture plates (Corning, USA), and then incubated in a dark, moist incubator at 28°C for 2 h. Haemocytes were collected by a cell scraper and washed twice with crab saline and centrifuged at 1200 r/min for 3 min to remove non-ingested latex beads. Haemocytes were fixed in a fixative (1% methanal and 1% sucrose), and observed under an inverted fluorescence microscope. Five random fields with more than one hundred cells in each well were counted and three wells were used for each replicate. The phagocytic activity was presented as a percentage of phagocytized haemocytes out of toal haemotyes.

### Comet assay

The alkaline comet assay was performed according to the method described in previous study [23] with a slight modification. Preheated microscope slides were coated with 100 μl of 0.8% normal melting agarose (NMA) and covered with a coverslip and then stored at 4°C immediately for 10 min to stabilise the first agarose layer. After collection of the haemocytes, 50 μl of 1% low melting agarose (LMA) was mixed with 25 μl of cell suspension, which was adjusted to 1× 10^6^ ml^-1^ and layered over the first agarose layer and then stored at 4°C for 25 min. Subsequently, slides were placed in freshly prepared cell lysing solution (2.5 mol/L NaCl, 100 mmol/L Na_2_EDTA, 10 mmol/L Tris–HCl, 1% Triton X-100 and 10% DMSO, pH 10) at 4°C for 20 min. After rinsing in phosphate buffer solution (PBS) twice, slides were placed in a horizontal electrophoresis chamber covered with alkaline electrophoresis buffer (300 mmol/L NaOH, 1 mmol/L EDTA, pH 13) for 20 min to allow for the unwinding of DNA. Electrophoresis was performed under 25 V and 300 mA for 20 min, and slides were gently removed and neutralised three times for 5 min in a neutralisation buffer (0.4 M Tris–HCl, pH 7.5). Finally, slides were stained with 20 μl of 0.1 mg/ml propidium iodide in a dark moist chamber for 5 min and observed under the inverted fluorescence microscope. Three slides were prepared for each replicate, and ten random fields with at least 100 cells from each slide were analysed using the Comet Assay Software Project (CASP), with the DNA damage expressed as comet ratio and percentage of DNA in tail. All procedures above were operated under yellow light.

### MN (Micronucleus) assay

The MN assay was performed according to the method of S. Barka [24]. The haemocytes collected in section 2.4 were fixed in a fixative (1% glutaraldehyde and 1% sucrose) for 30 min at 4°C and then dyed with Gimesa solution (Sigma, Germany) for 5 min and rinsed in distilled water 3 times. Slides were air-dried and observed under a Motic AE21 inverted microscope (Motic, Canada) with oil lens (10×100). At least one thousand cells with preserved cytoplasm were scored per slide to determine MN frequency. Three slides were observed for each replicate, and MN frequency was expressed as the number of cells containing MN/total cells.

### Statistical analyses

A probit analysis was used to estimate the LC_50_ values and their 95% confidence limits. The results in all figures are the average values of 3 replicates±standard error. The data were processed with the aid of the SPSS V21.0 software and statistically analysed by analysis of variance (ANOVA). The normality of data was tested by the Shapiro Wilks test for all bioassays and a homogeneity test of variances was performed, and a multiple-comparison Duncan test was used to determine significant differences between each group and the control. The concentrations, the time of exposure and their interactions in each biochemical response was analysed by two-way ANOVA, and P value <0.05 was considered significant.

## Results

### LC_50_ of avermectin on *E.sinensis*

First, to determine the toxicity of avermctin on crabs, we determined the LC_50_ of avermectin on *E*. *sinensis*. In this test, all crabs in the control group survived during the experiment. According to the results of the probit analysis, the 48-h and 96-h LC_50_ values of avermectin on *E*. *sinensis* were 1.663 mg/ L (95% confidence limits: 1.239–2.247 mg/L) and 0.954 mg/L (95% confidence limits: 0.692–1.228 mg/L), respectively, and the safe concentration was 0.182 mg/L.

### Avermectin inhibits activities of antioxidant and induce levels of oxidative products

**Fig 1:**
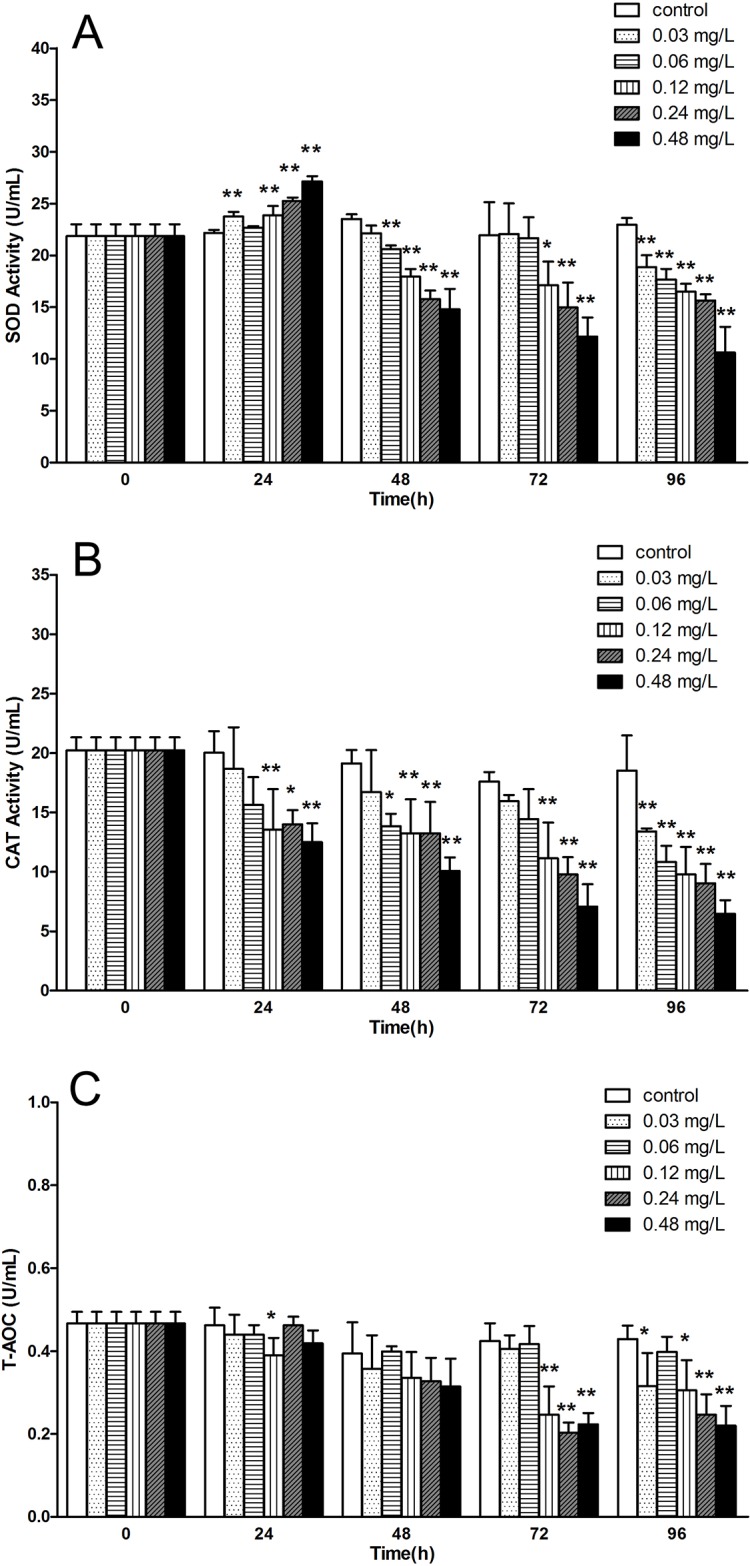
The variation of antioxidant activity in serum of *E.sinensis* exposed to different concentration of avermectin for 96 h. (A) superoxide dismutase (SOD) activity; (B) catalase (CAT) activity; (C) total antioxidant capacity (T-AOC). Note: asterisks (*) over the column indicate the significant difference between each experimental group and the control, one for *P*< 0.05 and two for <0.01, by using the ANOVA analysis. Values represent the mean ± SD of 3 independent samples, and error bars indicate standard deviation.

Next, we checked the activities of antioxidant and levels of oxidative products after different concentrations of avermectin treatment. SOD activities in all treatments increased significantly at 24 h except 0.06 mg/L, with a maximum of about 1.2 fold change comparing to control group. However, activities of SOD in all treatments dropped rapidly from 48 h. At 96 h, SOD activity in each group was significantly lower than control (P <0.01) (Fig 1A). CAT activities in high concentration groups (0.12, 0.24 and 0.48 mg/L) decreased significantly early at 24 h, and with the progress of exposure, there was significant difference (P <0.01) between control and each treatment with a dose- and time- dependent decrease (Fig 1B). Similar trend was found in the level of T-AOC that high concentration groups decreased significantly during the test (Fig 1C).

**Fig 2:**
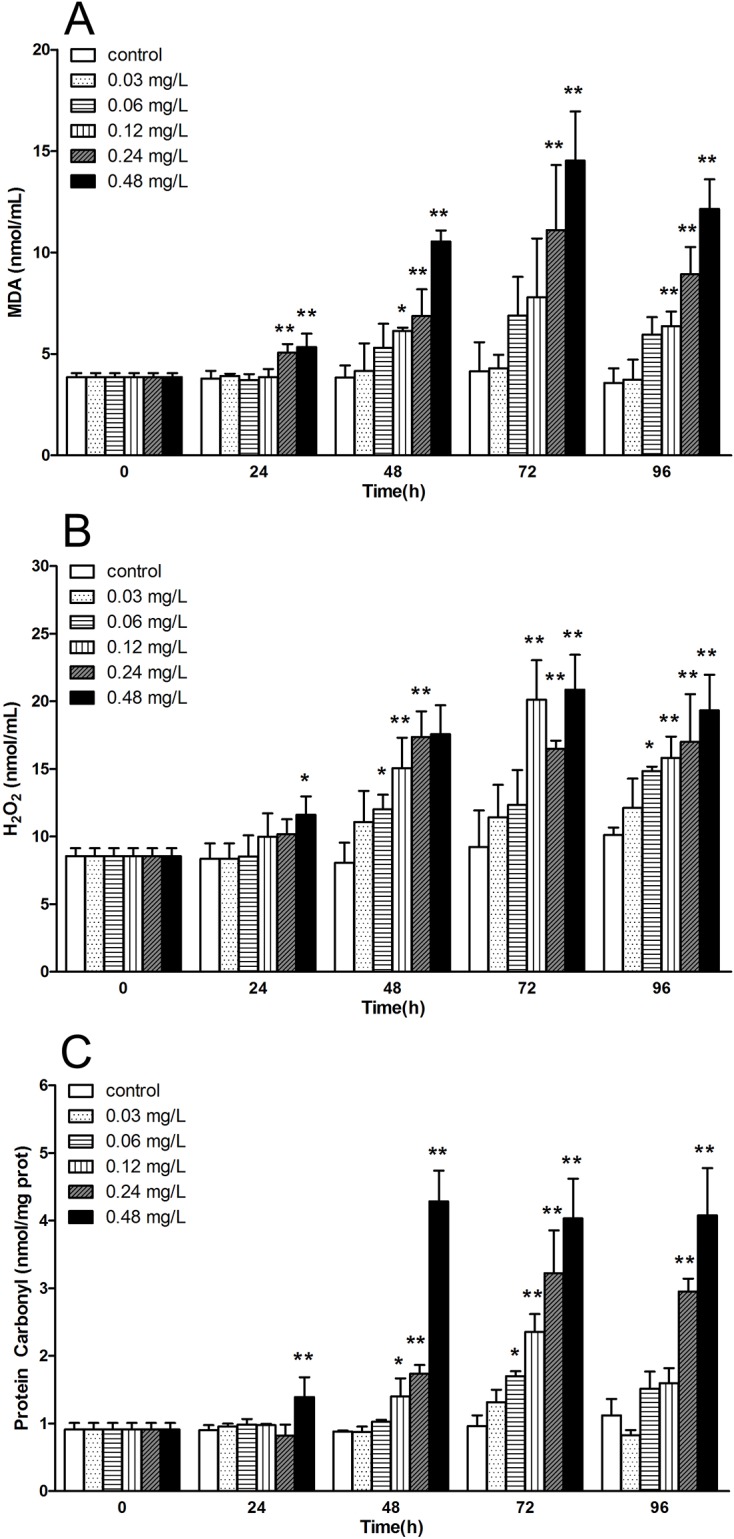
The variation of level of oxidative products in serum of *E.sinensis* exposed to different concentration of avermectin for 96 h. (A) malondialdehyde (MDA); (B) hydrogen peroxide (H_2_O_2_); (C) protein carbonyl. Note: asterisks (*) over the column indicate the significant difference between each experimental group and the control, one for *P*< 0.05 and two for <0.01, by using the ANOVA analysis. Values represent the mean ± SD of 3 independent samples, and error bars indicate standard deviation.

On the other hand, the level of MDA in serum increased gradually from 24 h and reached a peak at 72 h at concentrations of 0.24 and 0.48 mg/L, which were significantly higher than control. However, there was a slight recovery at 96 h (Fig 2A). Similar trend was observed in the variation of H_2_O_2_ levels. At 96 h, all treatments except concentration group of 0.03 mg/L were significantly higher than control (Fig 2B). In addition, no significant change of the level of protein carbonyl was observed at low concentration groups (0.03 and 0.06 mg/L) throughout the experiment, while they were approximately 2.6 and 3.6-fold higher than control at concentration of 0.24 and 0.48 mg/L, respectively (Fig 2C).

### Avermectin induces intracellular ROS production in haemocytes

**Fig 3:**
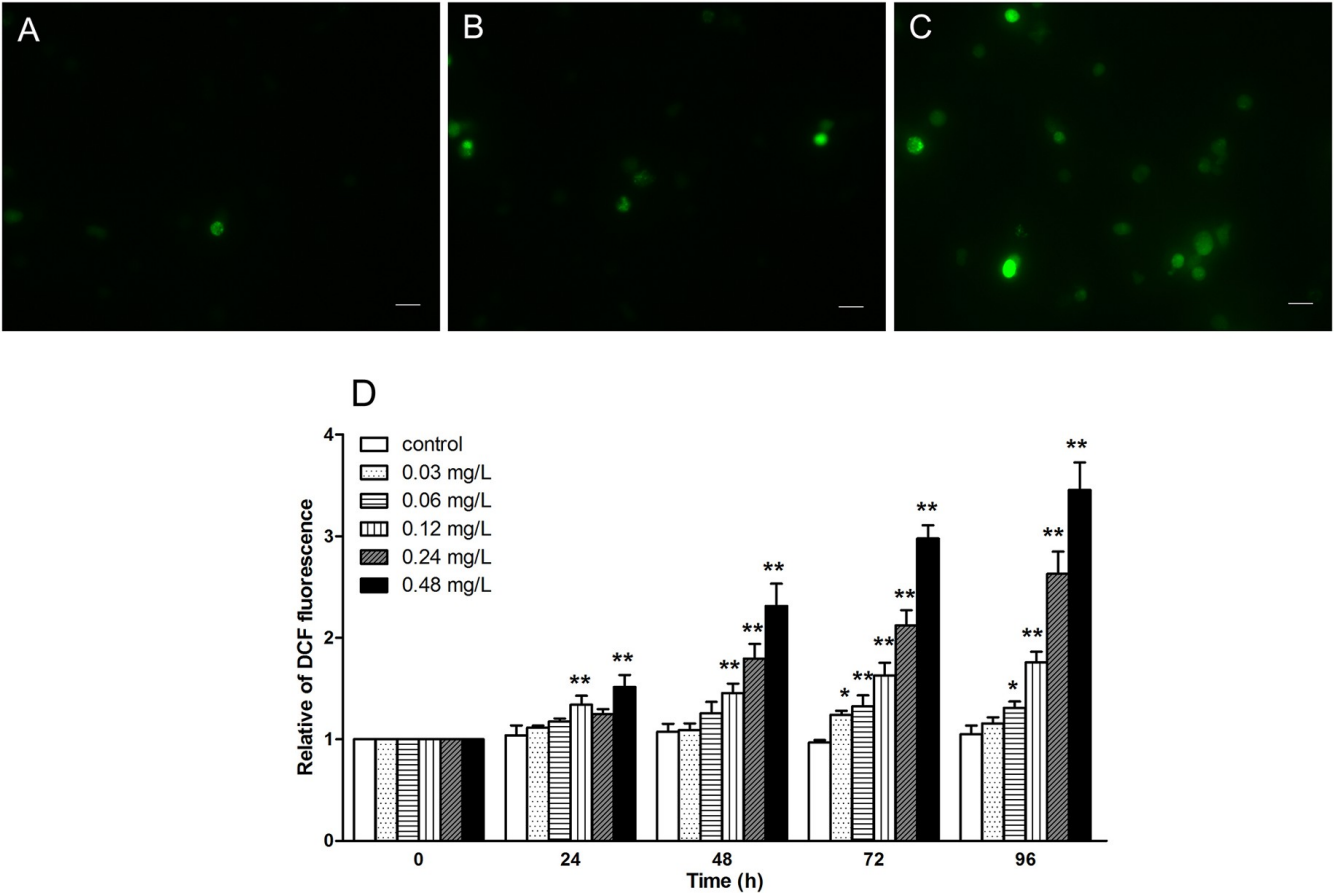
The generation of reactive oxygen species (ROS) induced by avermectin in haemocytes of *E.sinensis*. (A) haemocytes stained in DCFH-DA in control after 96 h exposure under a fluorescence microscope; (B) haemocytes in 0.06 mg/L after 96 h exposure; (C) haemocytes in 0.48 mg/L after 96 h exposure. Scale bar = 20μm. (D) relative of DCF fluorescence in each group calculated by the Image J software. Note: asterisks (*) over the column indicate the significant difference between each experimental group and the control, one for *P*< 0.05 and two for <0.01, by using the ANOVA analysis. Values represent the mean ± SD of 3 independent samples, and error bars indicate standard deviation.

Then, we measured the ROS level in haemocytes, since SOD and CAT activities are involved in ROS clearance. The effects of avermectin on the ROS generation is displayed in Fig 3. In comparison to treatment group, less staining cells could be observed, and numbers of staining cells rose in amount with increasing of concentration after avermectin exposure (Fig 3A, 3B and 3C). With progress of the test, generated ROS constantly increased with a dose-and time- dependent response in high concentration groups (Fig 3D).

### Avermectin inhibits phagocytic activity of haemocytes

**Fig 4:**
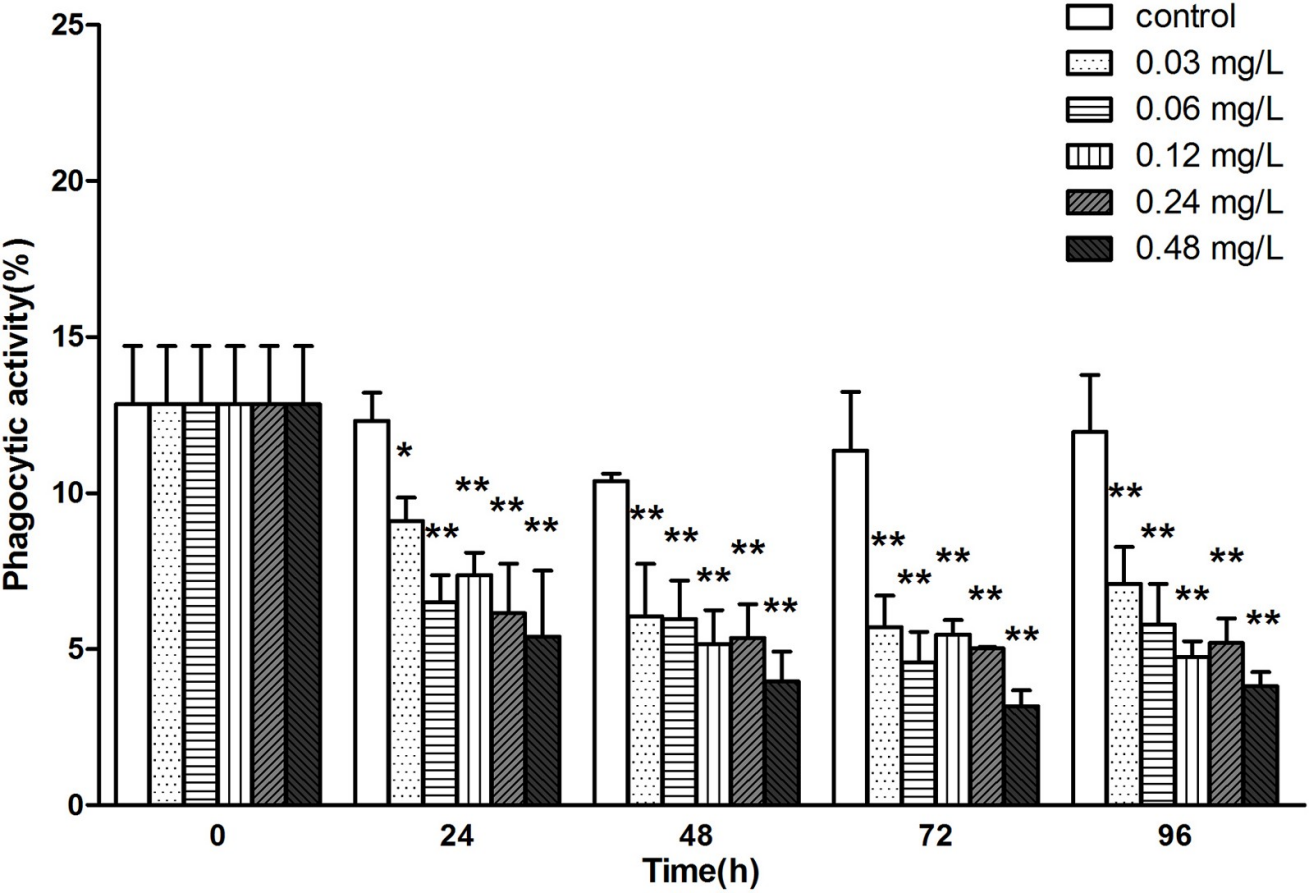
The variation of phagocytic activity of haemocytes in *E.sinensis* exposed to avermectin for 96 h. Note: asterisks (*) over the column indicate the significant difference between each experimental group and the control, one for *P*< 0.05 and two for <0.01, by using the ANOVA analysis. Values represent the mean ± SD of 3 independent samples, and error bars indicate standard deviation.

In addition, we also detected the phagocytic activity of haemocytes as a parameter of immune response. The phagocytic activities of haemocytes declined dramatically from 24 h.in all treatments compared to control group. The phagocytic activity remained at relatively low levels (half of the normal) at each concentration during the experiment and no recovery was found within the exposure period (Fig 4).

### Avermectin induces DNA damage in haemocytes

**Fig 5:**
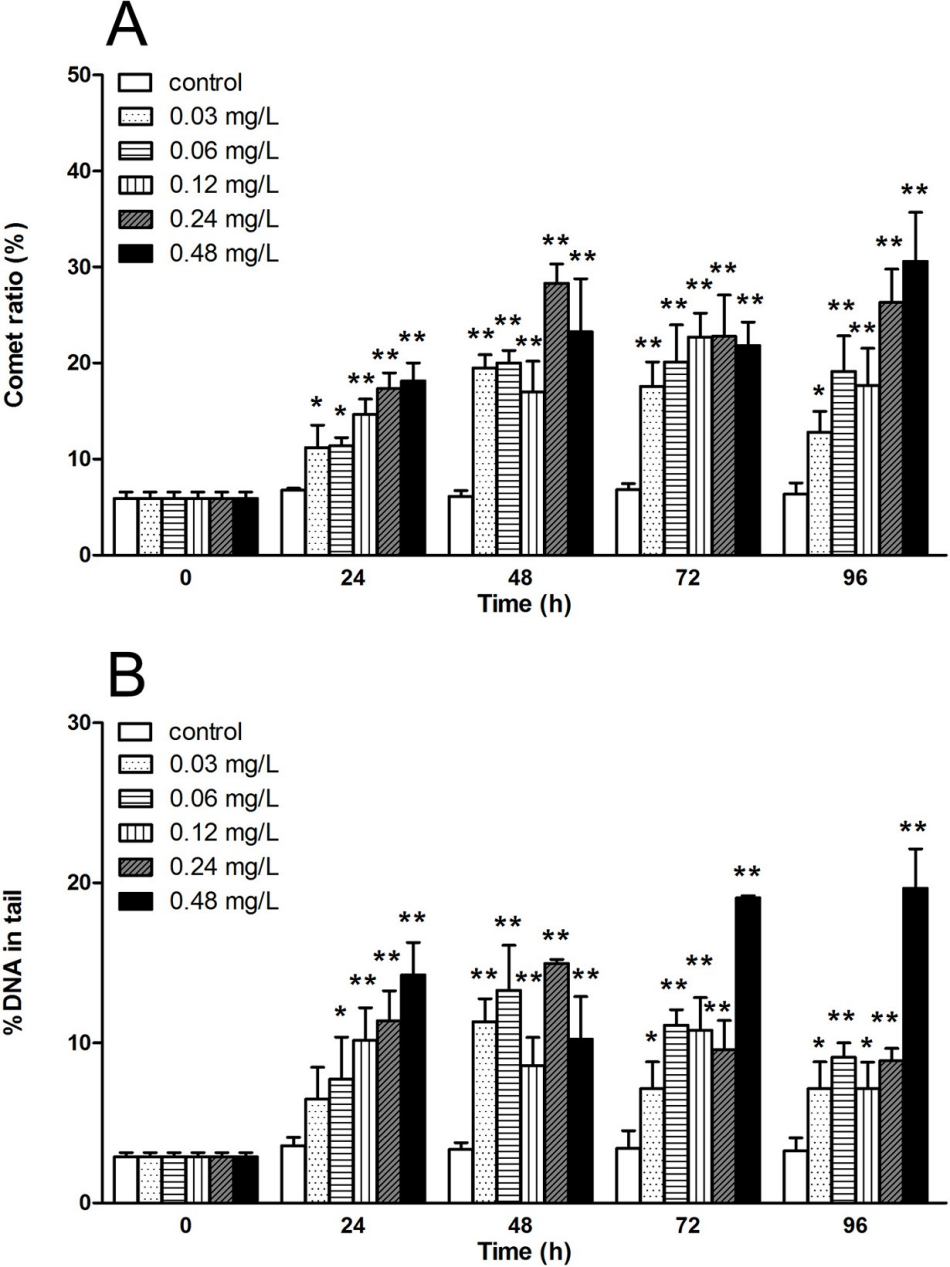
The DNA damage in haemocytes of *E.sinensis* exposed to avermectin for 96 h. (A) Comet ratio, which indicate the percentage of comet-positive cells in each group; (B) %DNA in tail in each group calculated by CASP software. Note: asterisks (*) over the column indicate the significant difference between each experimental group and the control, one for *P*< 0.05 and two for <0.01, by using the ANOVA analysis. Values represent the mean ± SD of 3 independent samples, and error bars indicate standard deviation.

On the other hand, a comet assay was performed to investigate the level of DNA damage under avermectin exposure. In control, most haemocytes were observed with a circular, intact nuclei, whereas different degree of comets in the exposure groups early at 24 h. More comets appeared with increase of avermectin concentration. The comet ratio and percentage of DNA in tail increased significantly at each concentration from 24 h (P< 0.05). In group of 0.48 mg/L, the comet ratio and percentage of DNA rose about 2.7 and 4.1 fold respectively, in comparison to control at 24 h. Although there was a slight recovery at 48 or 72 h, it continued to increase at 96 h. Even in group of 0.03 mg/L, the values of both comet ratio and percentage of DNA are 3.8 and 5.0 fold higher than control at 96 h.(Fig 5).

### Avermectin induces MN frequency in haemocytes

**Fig 6:**
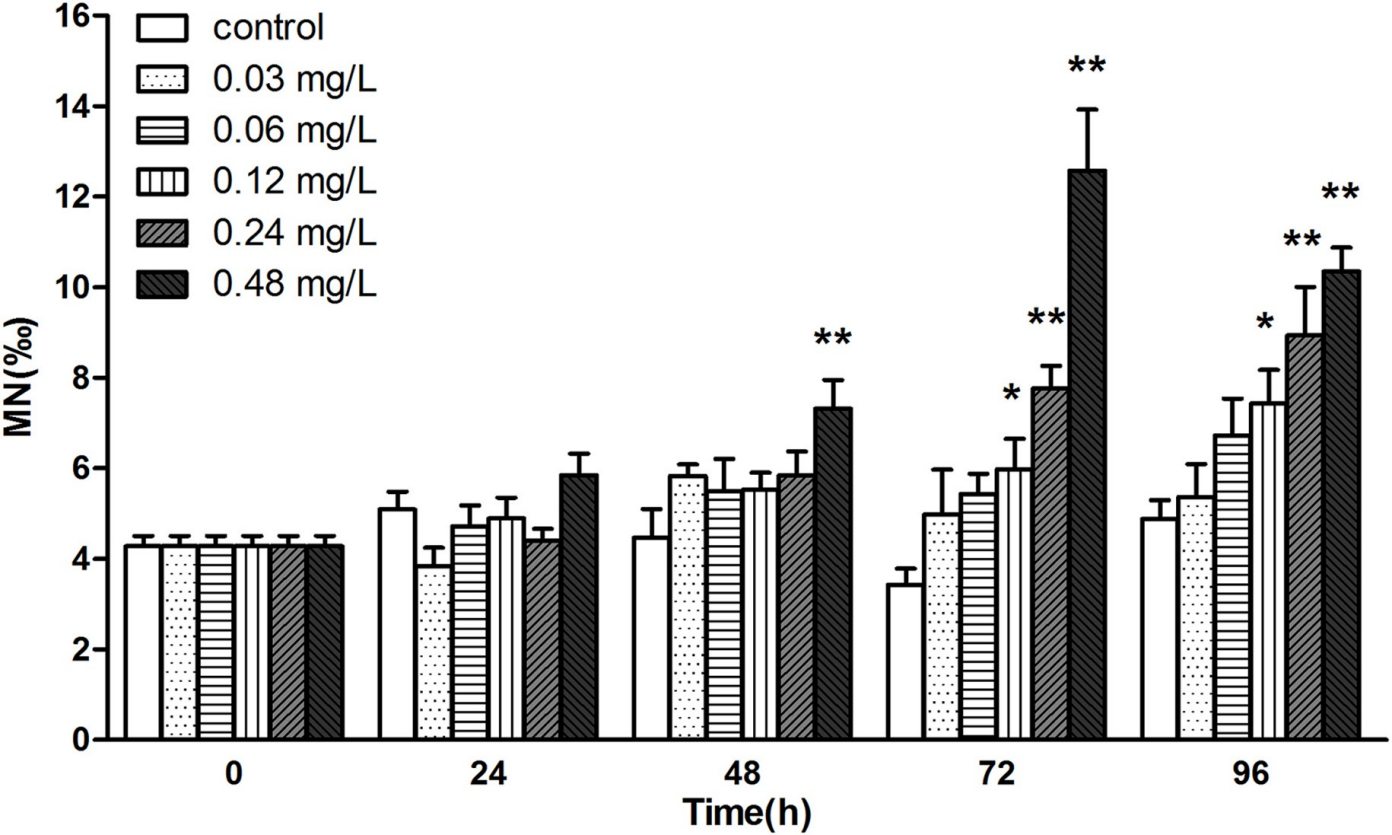
The variation of micronucleus (MN) frequency of haemocytes in *E.sinensis* exposed to avermectin for 96 h. Note: each column represent the percentage of cells containing micronucleus, asterisks (*) over the column indicate the significant difference between experimental group and the control, one for *P*< 0.05 and two for <0.01, by using the ANOVA analysis. Values represent the mean ± SD of 3 independent samples, and error bars indicate standard deviation.

In the end, we checked the MN frequency in haemocytes during the exposure. An obvious increase of MN frequency was observed at high concentrations especially at 0.48 mg/L from 48 h exposure. And at 96 h, there is a significant difference in concentration groups of 0.12, 0.24 and 0.48 mg/L compared to control (P< 0.05). However, no significant changes was observed at low concentrations during the exposure (Fig 6).

### Biochemical factorial analysis

According to the results from two-way ANOVA, a significant interaction between avermectin concentration and exposure time was found for all biomarkers involved (Table 1). All biomarkers investigated in this test showed a significant correlation between the concentration, exposure time and their cross combination. It indicated that both avermectin concentrations and exposure time have significant effects on the oxidative, immunological response and DNA damage in *E.sinensi*s, and all these biomarkers revealed its sensitivity in the assessment of toxicity induced by avermectin.

**Table 1:** Analysis of biochemical responses of *E.sinensis* under the avermectin exposure.

Biomarkers	Dose			Time			Dose*Time		
	Df	F	P	Df	F	P	Df	F	P
SOD	5	27.58	<0.0001*	4	70.98	<0.0001*	20	11.6	<0.0001*
CAT	5	30.48	<0.0001*	4	50.20	<0.0001*	20	2.473	0.0036*
T-AOC	5	14.2	<0.0001*	4	37.94	<0.0001*	20	3.256	0.0002*
MDA	5	43.63	<0.0001*	4	38.18	<0.0001*	20	6.118	<0.0001*
H_2_O_2_	5	30.42	<0.0001*	4	52.39	<0.0001*	20	4.037	<0.0001*
PC	5	124.9	<0.0001*	4	94.76	<0.0001*	20	19.98	<0.0001*
ROS	5	221.6	<0.0001*	4	208.4	<0.0001*	20	43.36	<0.0001*
AChE	5	119.9	<0.0001*	4	118.6	<0.0001*	20	12.96	<0.0001*
PA	5	37.51	<0.0001*	4	88.57	<0.0001*	20	2.815	0.0010*
CR	5	59.71	<0.0001*	4	90.89	<0.0001*	20	7.097	<0.0001*
%DNA	5	61.07	<0.0001*	4	66.79	<0.0001*	20	10.30	<0.0001*

PC: protein carbonyl; PA: phagocytic activity; CR: comet ratio;

* indicate a significant (P<0.05) effect on each biochemical response.

## Discussion

In spite of the numerous reports on the presence of avermectin in aquatic ecosystem and the toxicology to aquatic species, information about the toxic effects to macrocrustacean is still incomplete [7,6,25]. Particularly, in a co-culture system where avermectin is commonly used. In the present study, several parameters associated to oxidative stress, immune response and genotoxicity were measured comprehensively to investigate the potential toxic effect on *E*. *sinensis* induced by avermectin for the first time.

Avermectin is proved to be extremely toxic to aquatic species, for example, the 48 h- EC_50_ of avermectin for *Daphnia magna* is 0.25 μg/L [8] and the 96 h- LC_50_ for *Danio rerio* is 59 μg/L [26]. Study from Yin showed that avermectin is highly toxic to *Macrobrachium nipponense* with a 96 h- LC_50_ of 0.0533 mg/L [27]. However, in the present work, the 96 h- LC_50_ of avermectin on *E.sinensis* was 0.954 mg/L, which is much higher than the values in other aquatic organisms. It indicated that *E.sinensis* have a higher tolerance to avermectin exposure. Additionally, the half-life of avermectin in water is relatively short, which consequently minimises their eco-toxicological risk, and makes the practitioners to ignore its potential hazard. Nevertheless, from a previous study, the half-life of avermectin in paddy water could up to 4.5 d [25] and dwellers such as mitten crabs may suffer more than fishes under the avermectin exposure. On the other hand, avermectin could affect crabs through the runoff following crop protection in the area near by, or in a rice-crab or fish-crab co-culture systems for pest control. In this sense, the present study conducted an acute toxic test under several sublethal concentrations, including concentrations under the application dosage in planting region with 0.04 mg/L [9] or in rice field with 2.7 mg/L [19].

SOD and CAT are proved to participate in several physiological and metabolic reactions, especially in the clearance of free radical and prevention of biological molecular injury [28]. In the present study, SOD activities increased first at 24 h and then decreased with a time- and dose dependent response. However, no increase was found in CAT activity while it decreased gradually during the exposure at each concentration. These results were in accordance with the study from Chen et al [29] that SOD activities in common carp increased first and decreased significantly after 48 h exposure of avermectin at concentrations of 5.6 and 7.5 μg/L. Reports from Ma et al [30] also indicated that avermectin could induce SOD activity in freshwater snail *Physa acuta* following 24 h exposure but decreased at 96 h at concentrations ranging from 9.6 to 27.4 μg/L. However, the tendency of CAT variation in the snail was opposite to our results. Several studies involving the oxidative stress induced by pesticides including glyphosate [23], deltamethrin [31], imidacloprid [32] and eprinomectin [13] showed the inhibition of CAT activity in aquatic animals. This may due to the different tolerance to avermectin in different species, and in addition, the higher concentrations (more than 30 μg/L) in this test. The T-AOC, which represent the total level of antioxidation, offers many advantages and is considered as a useful tool for detecting oxidative stress phenomena in bodily fluids and tissues [33]. In this study, the T-AOC showed a similar trend with CAT activity that decreased remarkably at high concentrations. However, it varied much slower than SOD and CAT at low concentrations. We suggested that other antioxidants may play important roles in the antioxidative defense to remain the antioxidation system while SOD and CAT systems were mutilated by pesticide exposure, and this need to further investigation.

On the other hand, the levels of oxidative products including MDA, H_2_O_2_ and protein carbonyl were increased significantly especially at high concentrations. The results were in good consistent with studies in pigeon [34] and snail [30] that under the avermectin exposure, the level of MDA and protein carbonyl in tissues increased rapidly with time- and dose- dependent manners. In our previous study [35], H_2_O_2_ could be sensitive biomarkers in the assessment of deltamethrin exposure to *E.sinensis*, and in this work, it also sensitive to the exposure of avermectin at concentration above 0.03 mg/L. ROS play important roles in several intracellular physiological response like apoptosis by acting as a trigger of signaling molecules. The collapse of equilibrium of ROS production and elimination could induce cell injury, apoptosis or even necrosis [12]. In this study, a dose- and time- dependent increase of ROS generation was observed in haemocytes, which is consistent with the variation of oxidative products in serum as well as the DNA damage in haemocytes. It revealed that avermectin exposure brings about a significant increase of ROS in haemocytes of *E*. *sinensis*, to induce DNA damage following the antioxidative inhibition possibly caused by cell apoptosis.

Phagocytosis is commonly considered as one of the most important cellular immunity reactions to pathogen invasion in crustaceans [36]. The current study showed markedly inhibition of phagocytosis in *E.sinensis* during the exposure of avermectin, even at concentration of 0.03 mg/L. These results were in accordance with studies from Xian et al that phagocytic activity in haemocytes of *Penaeus monodon* decreased significantly when exposed to Cu^2+^ [37]. As a consequence, the irreversible inhibition in phagocytic activity during the acute exposure of avermectin may lead the host susceptible to pathogens.

The application of comet assays in the assessment of genotoxicity has been reported for many aquatic animals such as fishes and bivalves [38–40], but few reports were found in macrocrustaceans such as crabs and shrimps. In the present study, we performed the comet assay in haemocytes successfully to evaluate the genotoxicity of avermectin on *E*. *sinensis*. DNA damage was found early at 24 h and both comet ratio and %DNA in tail in all treatments were significantly increased, except %DNA at 0.03 mg/L. Then, a slight recovery was found at each concentration at 48 h or 72 h, following a increase at 96 h. These findings indicated that avermectin could induce significant DNA damage in haemocytes from *E*. *sinensis* even below the safe concentration (0.182 mg/L). The results are consistent with those of a previous study that avermectin could induce significant DNA damage in haemocytes in silkworms within 3 days but had a recovery from 5 d [41]. This may be due to the mechanism of repair of DNA. According to the data, comet ratio and %DNA in the tail could be sensitive and accurate parameters for the detection of DNA damage under low concentration of avermectin exposure.

The MN test was developed as a simple and practical in vivo cytogenetic screening method for mutagens [42]. In the present study, MN frequency increased significantly in high concentration groups from 48 h and rose gradually with the progress of exposure, except the recovery in group of 0.48 mg/L at 96 h. However, no induction was observed at concentrations of 0.03 and 0.06 mg/L during the exposure. Several previous study showed the lack of MN induction under low concentrations or at high concentrations within 48 h, when aquatic animals exposed to heavy metal or pesticides [24,43]. This could be due to the stimulated erythro-catertic function which lead to a slow replacement of erythrocytes or haemocytes, and their delayed appearance in blood circulation.

## Conclusion

In this study, a prominent toxic effect of avermectin on the Chinese mitten crab, *E*. *sinensis* was detected, for the antioxidative and immunological activity inhibition and ROS generation, as well as the DNA damage and MN formation in haemocytes even at concentration of 0.03 mg/L, which is lower than the safe concentration and also the application dosage in practice. It would increase the risk of pathogen invasion as the host is under a sub-lethal status, particularly in the high-occurrence seasons when avermectin is in a peak usage. Therefore, it suggested that not to use in aquatic water.

## Supporting information

S1 File Raw data of all tests in this study. (RAR)
